# Association of physical activity volume with the risks of nervous system diseases: a retrospective cohort study

**DOI:** 10.3389/fneur.2026.1804767

**Published:** 2026-04-08

**Authors:** Mingming Ye, Yibai Zhu, Kaiyun Xu, Li Li, Fangyuan Hu

**Affiliations:** 1 Emergency Department, Eastern Hepatobiliary Surgery Hospital, Shanghai, China; 2 Health Care Department, Eastern Hepatobiliary Surgery Hospital, Naval Medical University, Shanghai, China; 3 Faculty of Health Service, Naval Medical University, Shanghai, China

**Keywords:** incidence, nervous system diseases, physical activity volume, retrospective study, UK Biobank

## Abstract

**Background:**

Physical activity volume (PAV) has been linked to a wide range of health outcomes; however, its association with incident nervous system diseases remains incompletely understood. This study explored their relationship using data from UK Biobank.

**Methods:**

A retrospective cohort study was conducted involving 278,306 participants from the UK Biobank. PAV was quantified as metabolic equivalent of task (MET) minutes per week, derived from self-reported physical activity levels, and categorized into three groups. Incident cases of nervous system diseases were identified through ICD-10 codes obtained from hospital inpatient records, death registries, and self-reports. Cox proportional hazards models were employed to estimate hazard ratios (HRs) and 95% confidence intervals (CIs), adjusting for a series of covariates. Restricted cubic splines were applied to assess potential non-linear associations.

**Results:**

Women and individuals aged 60 years or older exhibited higher incidence rates of nervous system diseases. After multivariable adjustment, higher PAV was significantly associated with a lower risk of overall nervous system diseases (HR < 1). A non-linear dose–response relationship was observed, with the lowest risk occurring at a PAV level of 1,356 MET-min/week. Subgroup analyses indicated that elevated PAV conferred protective effects against several specific conditions. Conversely, higher PAV was associated with an increased risk of meningitis.

**Conclusion:**

Increased levels of physical activity are associated with a reduced risk of numerous nervous system diseases, with optimal protection observed at approximately 1,356 MET-min/week. These findings support the promotion of moderate-to-vigorous physical activity as a preventive strategy for neurological disorders, particularly among high-risk populations.

## 1 Introduction

The increasing global burden of nervous system diseases is notable, driven by aging populations. Nervous system diseases, including neurodegenerative diseases (1, 2), cerebrovascular diseases (e.g., stroke) (3), and other neurological disorders (4), have become leading causes of disability and death worldwide (5, 6). With the development of medical technique and pharmacological treatments, the treatments of nervous system diseases are continually evolving (7–9). While clinical treatment often only symptoms management, and some conditions need long-term rehabilitation, the role of exercise in the recovery of the nervous system remains significant (10). As we all know, exercise is a well-established protective factor for some diseases, such as diabetes (11), gastroesophageal reflux disease (12), and even some cancers. Besides, a growing body of evidence suggests that physical activity also contribute to brain health (10). Proposed mechanisms include enhanced cerebral blood flow, reduction of inflammation, improved vascular function, and the promotion of neurotrophic factors. Numerous studies have linked exercise to a reduced risk of individual nervous system diseases, particularly dementia and stroke (13). However, notable gaps in knowledge remain. Current studies either have focused on a single disease outcome or simply illustrate exercise condition using active vs. inactive. The quantitative relationship between the total volume of activity and nervous system disease is unclear (14). Understanding this dose–response relationship is crucial for informing precise public health recommendations.

Physical activity volume(PAV), the total amount of physical activity one performs, is regarded as the comprehensive measure of total exercise “dose” (15). It is a crucial concept in public health and the key factor linked to most health benefit, calculated by manipulating frequency, intensity, and time (16). In order to make sense of association between the volume of physical activity and the subsequent incidence of nervous system disease, we conducted a large-scale retrospective cohort study using date from UK Biobank to explore their relationships.

## 2 Materials and methods

### 2.1 Date source

The UK Biobank is a long-term prospective cohort study established to identify the determinants of disabling conditions and data collected from 22 health centers across the United Kingdom (17). At recruitment, participants completed a detailed touch-screen questionnaire which mainly focus on demographic, socioeconomic information, health behaviors and living environment situations were collected by specific measurements. More details about the UK Biobank can be accessed online (http://www.ukbio bank.ac.uk/). The ethical approval was granted by the research ethics committee (11/ NW/0382). Before the research, all participants provided written informed consent and we were permitted to use UK Biobank data under study number 99709. At the beginning, data from 502,428 participants were potentially available. After excluding UK Biobank removed (*n* = 430), missing data on key covariates (*n* = 90,935), with no PAV data (*n* = 86,107), previous diagnosis of Nervous System Diseases at baseline (*n* = 46,650), data from a total of 278,306 eligible participants, aged from 40 to 69 at recruit in UK Biobank was used in our study. The participants were enrolled between 2006 and 2010, and the follow-up completed on March 31, 2025.

### 2.2 Calculation of PAV

PAV is the representation of the metabolic equivalent of tasks, and calculated by each participant’s weekly physical activity volume. In this study, the PAV was only used to evaluated physical exercise activity and calculated based on the 2011 Compendium of Physical Activities (18). As the physical activity intensity is usually divided into vigorous, moderate, and light, and each category is assigned eight, five, and three METs. According to the minimum time consumed for each category and its frequencies, the PAV was finally calculated as follows: (8 METs×20 min × times) + (5 METs×30 min × times) + (3 METs×30 min × times). Then, PAV was divided into the following three groups, which, respectively, is <500 group, 500–1,000 group, and ≥1,000 group.

### 2.3 Ascertainment of nervous system disease incidence

The incident Nervous System disease events was identified in accordance with the ‘first occurrence’ data fields that the UK biobank generated that map the clinical codes from primary care, hospital inpatient admissions, death records, and self-reported medical conditions to 3-digit code of International Classification of Disease (ICD-10) and provide, for each participant, the date that code first occurred in any source.

### 2.4 Covariates

The selection of covariates in our study reference to a review of several published studies and Global Burden Disease report (19). To mitigate the influence of potential confounding effects, the following five types covariates were considered in multivariable models: Demographic information (Sex, Age, Ethnic background, BMI and MET score), Socioeconomic Status (Employment status and University education), Medical History (Diabetes, Hypertension and Cancer), Health Behaviors (Alcohol drinker status, Smoking status, Sleep duration, Time watch TV, Time use computer, Coffee intake, Tea intake and IPAQ activity group) and Living Environment (PM2.5, PM10, Greenspace percentage in 300 m, Nitrogen dioxide and Nitrogen oxides). Among these, Sleep duration, Time watch TV and Time use compute were self-reported as the number of hours per day or the times per day. BMI or Body Mass Index was calculated using the internationally recognized formula: weight (kg) divided by height (cm) squared. IPAQ (International Physical Activity Questionnaire) is a self-administered questionnaire that was used to assess the physical activity in the past 7 days. As regular PA was defined as performing at least 30 min of moderate PA at least five times a week, we categorized each participant’s weekly PA levels based on that. In this study, we used IPAQ to evaluate the physical activity intensity. Furthermore, considering diabetes, hypertension and cancer are major components of the global burden of disease, this study included these three conditions based on the first reported date in the first occurrence dataset and the date of attending the assessment center.

### 2.5 Statistical analysis

Statistical analyses used SAS (version 9.4, SAS Institute Inc., Cary, NC, United States) and R (version 4.4.1), with statistical significance at *p* < 0.05 using t-tests for continuous variables and chi-square for categorical variables. Cox proportional hazards regression models were used to explore the associations between PAV score and total nervous system disorder incidence, and hazard ratios (HRs) and 95% confidence intervals (CIs) were shown. The four models were tested: (1) Model 1 was adjusted for demographic information; (2) Model 2 was adjusted for Model 1 plus socioeconomic status; (3) Model 3 was adjusted for model 2 plus medical history factors; (4) Model 4 further adjusted for health behaviors factors; and (4) Model 5 finally adjusted for living environment factors.

We used restricted cubic spline (RCS) regression to explore potential non-linear associations between PAV score total nervous system disorder incidence. The subgroup analysis was used to further explore associations between PAV score and specific nervous system disorder incidence.

## 3 Results

### 3.1 Characteristics of the study population

Data from 502,428 participants were available at beginning, after excluding UK Biobank removed (*n* = 430), missing data on key covariates (*n* = 90,935), with no PAV data (*n* = 86,107), previous diagnosis of Nervous System Diseases at baseline (*n* = 46,650), data from a total of 278,306 eligible participants in UK Biobank was used in our study.

Among UK biobank participants, a nearly equal gender distribution was shown, participants were almost white (94.8%), and age below 60 were162000 (58.2%). According to each participant’s PAV score, the number of participant in each group were, respectively, 40,780 (MTE < 500 group), 41,976 (500–1,000 group), 195,550 (MTE ≥ 1,000 group). Compared with those whose PAV score was above 1,000, participants in PAV < 500 group tended to be younger, possess a higher BMI, exhibit higher levels of education, and sleep less, smoke more. Additionally, participants in this group spent more time in watching TV and using computer. Furthermore, they also had a higher proportion of Diabetes and Hypertension than the other two groups. While no difference was shown when compared the ratio of Cancer. The characteristics of study participants in the primary overall and by the divided group were presented in Table 1.

**Table 1:** Characteristics of study population in UK Biobank.

Characteristics	PAV <500 group (40780) *n* (%)	PAV 500–1,000 group (41976) *n* (%)	PAV ≥1,000 group (195550) *n* (%)	Total (278306) *n* (%)	*p*-value
Sex					<0.0001
Male	20,502 (50.3%)	19,716 (47.0%)	96,406 (49.3%)	136,624 (49.1%)	
Female	20,278 (49.7%)	22,260 (53.0%)	99,144 (50.7%)	141,682 (50.9%)	
Age at recruitment					<0.0001
<60	25,799 (63.3%)	25,068 (59.7%)	111,133 (56.8%)	162,000 (58.2%)	
≥60	14,981 (36.7%)	16,908 (40.3%)	84,417 (43.2%)	116,306 (41.8%)	
BMI(kg/m2)					<0.0001
<18.5	404 (1.0%)	346 (0.8%)	1,600 (0.8%)	2,350 (0.8%)	
18.5–20	593 (1.5%)	698 (1.7%)	3,864 (2.0%)	5,155 (1.9%)	
20–25	9,925 (24.3%)	12,607 (30.0%)	66,937 (34.2%)	89,469 (32.1%)	
25–30	16,801 (41.2%)	18,037 (43.0%)	85,241 (43.6%)	120,079 (43.1%)	
≥30	13,057 (32.0%)	10,288 (24.5%)	37,908 (19.4%)	61,253 (22.0%)	
Ethnic background					<0.0001
White	38,142 (93.5%)	39,599 (94.3%)	186,182 (95.2%)	263,923 (94.8%)	
African	359 (0.9%)	331 (0.8%)	1,226 (0.6%)	1916 (0.7%)	
Asian	1,213 (3.0%)	1,046 (2.5%)	3,697 (1.9%)	5,956 (2.1%)	
Mixed	252 (0.6%)	252 (0.6%)	1,174 (0.6%)	1,678 (0.6%)	
Other ethnic group	814 (2.0%)	748 (1.8%)	3,271 (1.7%)	4,833 (1.7%)	
Current employment status					<0.0001
Employment	27,312 (67.0%)	26,821 (63.9%)	116,083 (59.4%)	170,216 (61.2%)	
Unemployment	3,574 (8.8%)	2,674 (6.4%)	12,566 (6.4%)	18,814 (6.8%)	
Retired	9,894 (24.3%)	12,481 (29.7%)	66,901 (34.2%)	89,276 (32.1%)	
University education					<0.0001
No	26,121 (64.1%)	25,455 (60.6%)	127,080 (65.0%)	178,656 (64.2%)	
Yes	14,659 (35.9%)	16,521 (39.4%)	68,470 (35.0%)	99,650 (35.8%)	
Alcohol drinker status					<0.0001
Current	37,521 (92.0%)	39,307 (93.6%)	183,436 (93.8%)	260,264 (93.5%)	
Previous	1,421 (3.5%)	1,149 (2.7%)	5,523 (2.8%)	8,093 (2.9%)	
Never	1838 (4.5%)	1,520 (3.6%)	6,591 (3.4%)	9,949 (3.6%)	
Smoking status					<0.0001
Current	4,941 (12.1%)	4,066 (9.7%)	18,037 (9.2%)	27,044 (9.7%)	
Previous	13,804 (33.9%)	14,340 (34.2%)	69,671 (35.6%)	97,815 (35.1%)	
Never	22,035 (54.0%)	23,570 (56.2%)	107,842 (55.1%)	153,447 (55.1%)	
Sleep duration (Hours/day)					<0.0001
<7	10,391 (25.5%)	9,643 (23.0%)	44,471 (22.7%)	64,505 (23.2%)	
≥7	30,389 (74.5%)	32,333 (77.0%)	151,079 (77.3%)	213,801 (76.8%)	
Diabetes					<0.0001
No	35,839 (87.9%)	38,098 (90.8%)	181,052 (92.6%)	254,989 (91.6%)	
Yes	4,941 (12.1%)	3,878 (9.2%)	14,498 (7.4%)	23,317 (8.4%)	
Hypertension					<0.0001
No	24,156 (59.2%)	26,149 (62.3%)	126,220 (64.5%)	176,525 (63.4%)	
Yes	16,624 (40.8%)	15,827 (37.7%)	69,330 (35.5%)	101,781 (36.6%)	
Cancer					0.5145
No	31,203 (76.5%)	32,225 (76.8%)	150,139 (76.8%)	213,567 (76.7%)	
Yes	9,577 (23.5%)	9,751 (23.2%)	45,411 (23.2%)	64,739 (23.3%)	
Coffee intake (Cups/day)					<0.0001
Not intake	12,213 (29.9%)	11,671 (27.8%)	54,193 (27.7%)	78,077 (28.1%)	
1	7,473 (18.3%)	8,583 (20.4%)	41,369 (21.2%)	57,425 (20.6%)	
2	7,033 (17.2%)	8,062 (19.2%)	38,618 (19.7%)	53,713 (19.3%)	
3	5,052 (12.4%)	5,315 (12.7%)	24,446 (12.5%)	34,813 (12.5%)	
4	3,586 (8.8%)	3,761 (9.0%)	16,411 (8.4%)	23,758 (8.5%)	
5	2,411 (5.9%)	2,106 (5.0%)	9,492 (4.9%)	14,009 (5.0%)	
≥6	3,012 (7.4%)	2,478 (5.9%)	11,021 (5.6%)	16,511 (5.9%)	
Tea intake (Cups/day)					<0.0001
Not intake	8,078 (19.8%)	7,322 (17.4%)	32,769 (16.8%)	48,169 (17.3%)	
1	3,798 (9.3%)	3,724 (8.9%)	16,776 (8.6%)	24,298 (8.7%)	
2	5,750 (14.1%)	6,385 (15.2%)	28,768 (14.7%)	40,903 (14.7%)	
3	5,912 (14.5%)	6,499 (15.5%)	29,870 (15.3%)	42,281 (15.2%)	
4	5,486 (13.5%)	6,005 (14.3%)	28,458 (14.6%)	39,949 (14.4%)	
5	4,335 (10.6%)	4,599 (11.0%)	22,483 (11.5%)	31,417 (11.3%)	
≥6	7,421 (18.2%)	7,442 (17.7%)	36,426 (18.6%)	51,289 (18.4%)	
Time watch TV (Hours/day)					<0.0001
<1	3,140 (7.7%)	3,476 (8.3%)	16,691 (8.5%)	23,307 (8.4%)	
1	4,978 (12.2%)	5,772 (13.8%)	27,764 (14.2%)	38,514 (13.8%)	
2	10,549 (25.9%)	11,718 (27.9%)	55,760 (28.5%)	78,027 (28.0%)	
3	9,503 (23.3%)	9,795 (23.3%)	45,848 (23.4%)	65,146 (23.4%)	
4	6,331 (15.5%)	6,307 (15.0%)	29,465 (15.1%)	42,103 (15.1%)	
≥5	6,279 (15.4%)	4,908 (11.7%)	20,022 (10.2%)	31,209 (11.2%)	
Time use computer (Hours/day)					<0.0001
<1	17,276 (42.4%)	18,425 (43.9%)	91,329 (46.7%)	127,030 (45.6%)	
1	11,633 (28.5%)	12,818 (30.5%)	61,116 (31.3%)	85,567 (30.7%)	
2	5,889 (14.4%)	5,870 (14.0%)	25,118 (12.8%)	36,877 (13.3%)	
3	2,178 (5.3%)	1912 (4.6%)	7,607 (3.9%)	11,697 (4.2%)	
4	1,259 (3.1%)	1,025 (2.4%)	3,942 (2.0%)	6,226 (2.2%)	
≥5	2,545 (6.2%)	1926 (4.6%)	6,438 (3.3%)	10,909 (3.9%)	
IPAQ activity group					<0.0001
Low	39,524 (96.9%)	8,807 (21.0%)	1,222 (0.6%)	49,553 (17.8%)	
Moderate	1,256 (3.1%)	33,169 (79.0%)	78,983 (40.4%)	113,408 (40.7%)	
High	0 (0%)	0 (0%)	115,345 (59.0%)	115,345 (41.4%)	
PM2.5*	9.92 [8.2, 21.3]	9.91 [8.17, 20.1]	9.90 [8.17, 20.2]	9.91 [8.2, 21.3]	<0.0001
PM10*	16.0 [11.8, 30.7]	16.0 [11.8, 26.3]	16.0 [11.8, 31.4]	16.0 [11.8, 31.4]	<0.0001
Greenspace percentage in 300m*	29.8 [0.3, 99.2]	29.3 [0.4, 99.1]	29.8 [0.2, 99.2]	29.7 [0.2, 99.2]	<0.0001
Nitrogen dioxide*	26.2 [12.9, 97.1]	26.2 [12.9, 108]	26.0 [12.9, 108]	26.0 [12.9, 108]	<0.0001
Nitrogen oxides*	42.3 [19.7, 247]	42.0 [19.7, 265]	41.9 [19.7, 266]	41.9 [19.7, 266]	<0.0001

^*^Median [Min, Max].

### 3.2 Relationships of PAV score and total nervous system disorder incidence

As shown in Table 2, negative associations between PAV score and the incidence of nervous system diseases were shown (HR < 1). Consistently, after adjusted the potential confounders of Socioeconomic Status (Employment status and University education) and Health Behaviors factors (Alcohol drinker status, Smoking status, Sleep duration, Time watch TV, Time use computer, Coffee intake, Tea intake and IPAQ activity group), the association seemed somewhat strengthened in Grouped PAV score. Whereas, after the adjustment of Socioeconomic Status (Employment status and University education) in Continuous PAV score, the association was insignificant, which might be caused by statistical difference. Additionally, adjusting the potential confounders of Medical History (Diabetes, Hypertension and Cancer) and Living Environment (PM2.5, PM10, Greenspace percentage in 300 m, Nitrogen dioxide and Nitrogen oxides), the incidence of total nervous system disorder seemed decrease. The *p* values for trend were all below 0.05 in above situations except in model 2 for Continuous PAV score.

**Table 2:** Associations between PAV score and total nervous system disorder incidence.

Model	Grouped PAV score		Continuous PAV score
	HR (95% CI)	P_Cox-regression_	P_Cox-regression_
Model 1	0.942 (0.929,0.954)	<0.001	0.003
Model 2	0.931 (0.919,0.944)	<0.001	0.497
Model 3	0.953 (0.941,0.967)	<0.001	<0.001
Model 4	0.945 (0.925,0.966)	<0.001	<0.001
Model 5	0.946 (0.926,0.967)	<0.001	<0.001

Model 1: Adjust Sex, Age at recruitment, Ethnic background, BMI and MET score.

Model 2: Adjust Variables in Model 1 plus Employment status and University education.

Model 3: Adjust Variables in Model 2 plus Diabetes, Hypertension and Cancer.

Model 4: Adjust Variables in Model 3 plus Alcohol drinker status, Smoking status, Sleep duration, Time watch TV, Time use computer, Coffee intake, Tea intake and IPAQ activity group.

Model 5: Adjust Variables in Model 4 plus PM2.5, PM10, Greenspace percentage in 300 m, Nitrogen dioxide and Nitrogen oxides.

**Figure 1:**
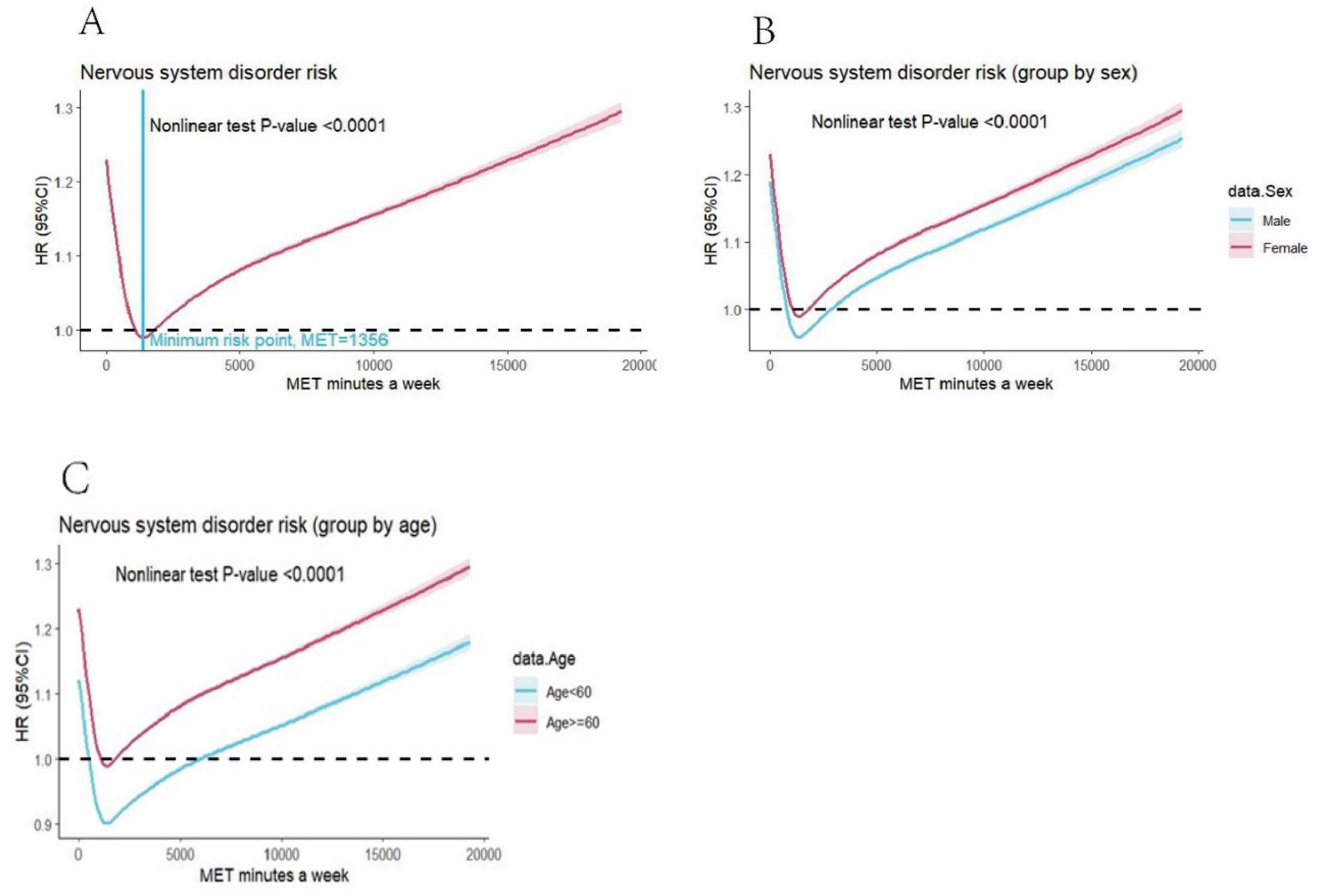
Restricted cubic spline for testing the hypothesis of nonlinear correlation between MET score and total nervous system disorder incidence in **(A)**. Total population, **(B)**. Population grouped by sex, **(C)**. Population grouped by age.

Non-linear relationship between the score of PAV and incidence of nervous system disorder after adjusting potential confounding factors. The risk of nervous system disorder was lowest when the score of PAV was 1,356. Considering the influence of gender and sex, female had higher incidence of Nervous System Diseases and population age above 60 were more likely had Nervous Diseases. More details were presented in Figure 1.

### 3.3 Relationships of PAV score and specific nervous system disorder incidence

**Figure 2:**
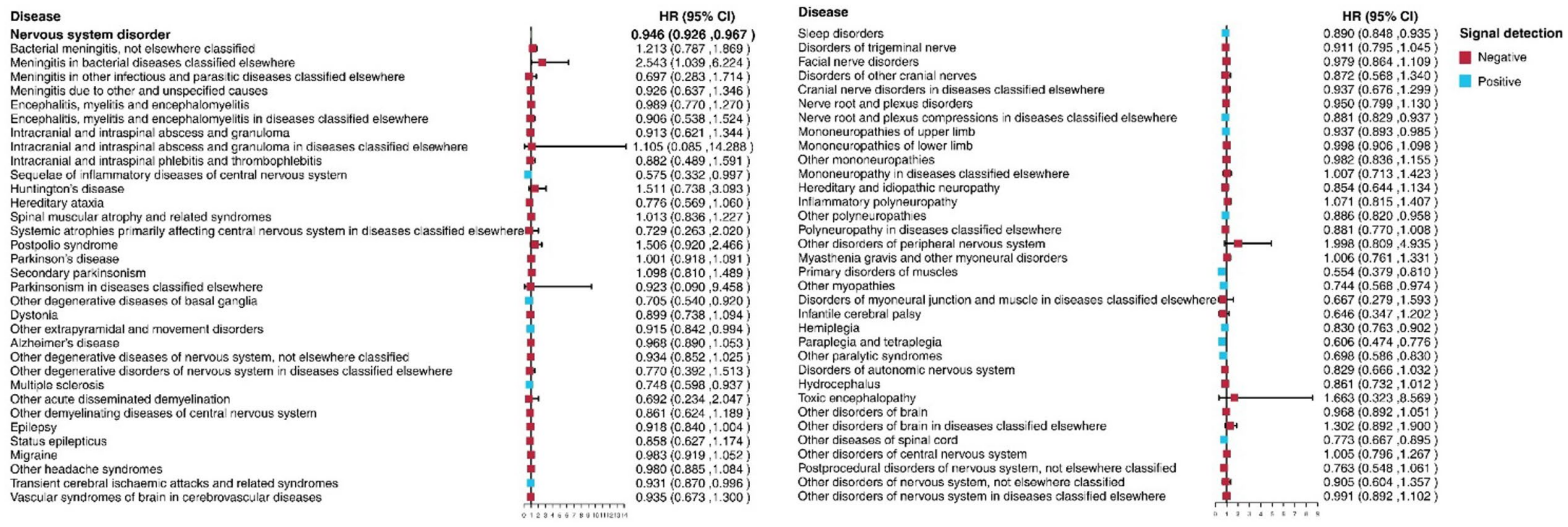
Associations between PAV score and specific nervous system disorder incidence.

Subgroup analysis was used to explored the relationships between PAV and some specific nervous system diseases. As shown in Figure 2, PAV had no influence on most nervous system diseases. While it was a risk factor to Meningitis in bacterial diseases classified elsewhere. Additionally, PAV played an protective effect in the following nervous system diseases: other degenerative disease of basal ganglia, other extrapyramidal and movement disorders, multiple sclerosis, transient cerebral ischaemic attacks and related syndromes, sleep disorder, nerve root and plexus compressions in diseases classified elsewhere, mononeuropathies of upper limb, other polyneuropathies, primary disorders of muscles, other myopathies, hemiplegia, paraplegia and tetraplegia, other paralytic syndromes, other disease of spinal cord.

**Figure 3:**
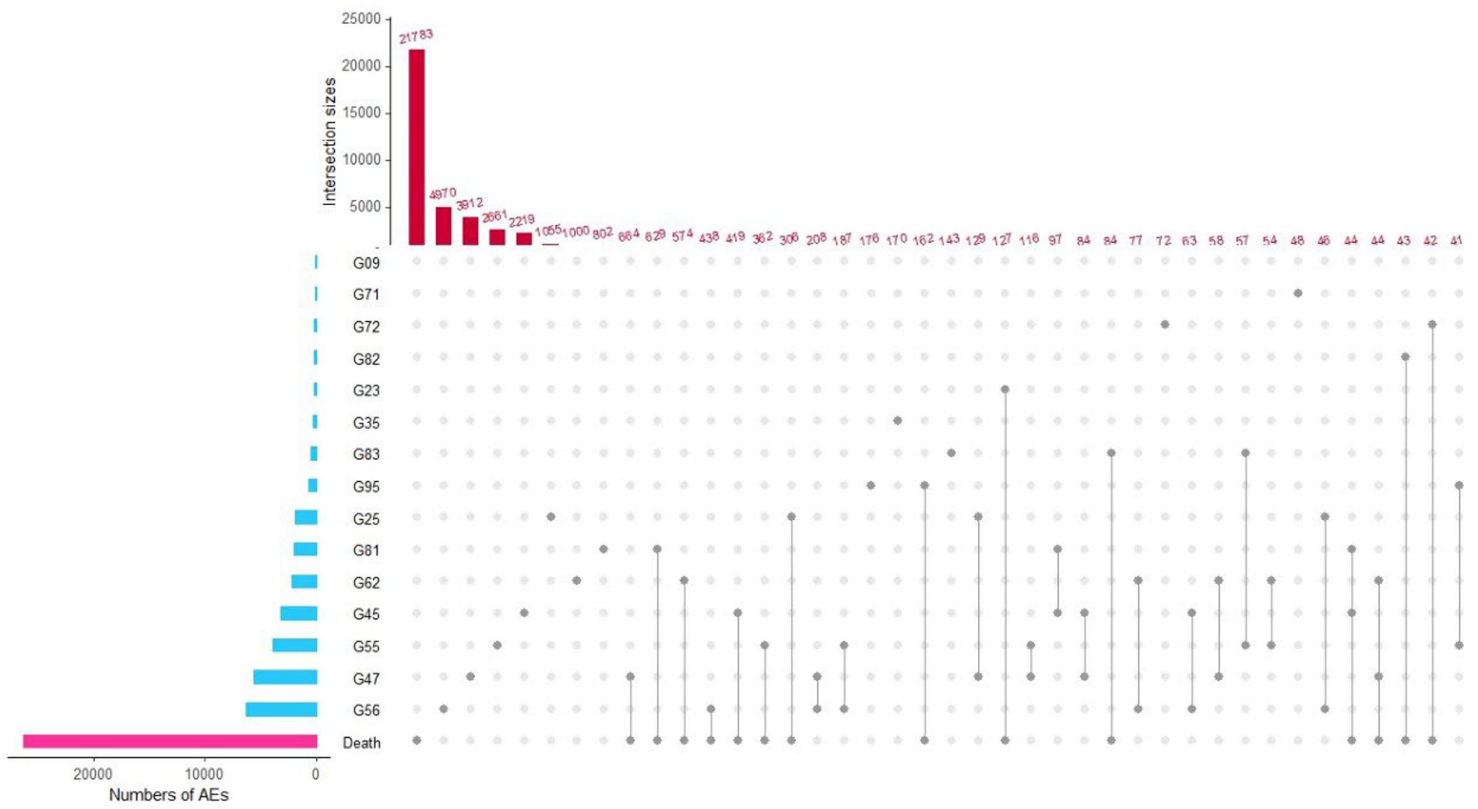
UpSet plots for signal-positive nervous system disorders and death intersection. G09, Sequelae of inflammatory diseases of central nervous system; G23, Other degenerative diseases of basal ganglia; G25, Other extrapyramidal and movement disorders; G35, Multiple sclerosis; G45, Transient cerebral ischaemic attacks and related syndromes; G47, Sleep disorders; G55, Nerve root and plexus compressions in diseases classified elsewhere; G56, Mononeuropathies of upper limb; G62, Other polyneuropathies; G71, Primary disorders of muscles; G72, Other myopathies; G81, Hemiplegia; G82, Paraplegia and tetraplegia; G83, Other paralytic syndromes; G95, Other diseases of spinal cord.

Furthermore, we explored the death ratio of those nervous system diseases to which PAV score showed as protective factors. The detailed death ratio of single nervous system disease and simultaneous two neurological disorders was shown in Figure 3. It shown that the top five death ratio of nervous system diseases are Mononeuropathies of upper limb, Sleep disorders, Nerve root and plexus compressions in diseases classified elsewhere, Transient cerebral ischaemic attacks and related syndromes, and Other extrapyramidal and movement disorders. While participants who simultaneous suffering two different types of nervous system diseases, the death odds of those were no increased.

## 4 Discussion

Regular physical exercise, encompassing both aerobic and resistance training, is widely acknowledged as a fundamental component of healthy lifestyle behaviors (20). Its benefits extend across multiple domains of health, particularly in promoting neurological well-being (21).

Physical exercise has been demonstrated to significantly reduce the risk of various neurodegenerative diseases (10), as well as autoimmune and inflammatory neurological disorders, such as multiple sclerosis and demyelinating diseases (22). The vascular protective effects of exercise are particularly noteworthy. These include enhanced cerebral blood flow, improved vascular function, and the promotion of neurotrophic factors such as Brain-Derived Neurotrophic Factor (BDNF) (23). These factors contribute to increased neural plasticity and a reduction in the production of inflammatory mediators, thereby attenuating neuroinflammation. Furthermore, the observed low prevalence of neuromuscular diseases and neurological sequelae—such as hemiplegia, paraplegia, and muscle paralysis—may be partially attributed to selection bias, as individuals with such conditions may be physically unable to engage in regular exercise and thus are underrepresented in the study population (24, 25).

The study also reveals that increased physical activity does not always equate to greater health benefits. Instead, an “optimal activity volume” appears to exist. It is in accordance with the recommendation of WHO about the physical activity intensity in different population (22). Beyond this threshold, the protective effects may plateau or even diminish, underscoring the importance of recommending appropriate exercise levels. Moreover, the incidence of nervous system diseases exhibits sexual dimorphism. The higher incidence rates of neurological diseases observed in women, potentially influenced by hormonal differences and variations in social behaviors. Same phenomenon also appears in other diseases, such as autoimmune diseases (26). The elevated risk among older adults further highlights the need for tailored preventive exercise interventions within this population.

Moderate-to-high-intensity physical activity (PAV) demonstrates a protective effect in the majority of neurological diseases. However, it may be ineffective or even harmful in certain infectious or inflammatory conditions, such as meningitis and nervous system abscess. This may be attributed to the investigation was conducted in the acute stage of diseases, in accordance with the suggestion that absolutely forbidding physical activity in outburn stage of meningitis (27, 28). This result highlights the necessity for disease-specific analyses.

Additionally, this study demonstrated the influence of living environment, which is coordinated with finds of one systematic review which explores the association between greenness exposure and nervous system disease risk and suggests further research focus more attention on the role greenness exposure plays in various nervous system diseases (29). Some previous studies have reported the increasing mortality of circulatory diseases and respiratory diseases with the exposure of PM_2.5_ (30).

Despite its strengths, this study has several limitations. First, physical activity levels were determined based on baseline health examination data, which may not fully reflect actual activity levels and may lack correlation with objective standard measurements. Due to the voluntary nature of participation, some participants lacked complete diagnostic data. Second, the classification of neurological diseases presents a complex challenge. Etiological and anatomical classifications often overlap, and diagnostic criteria vary across studies. The heterogeneity in disease severity may also introduce variability in the results. Third, there may be a considerable time interval between the health examination and the diagnosis of neurological diseases. Self-reported questionnaires are prone to recall bias. Fourth, the voluntary participation in health check-ups may lead to selection bias. Fifth, as an observational study, the observed associations may not reflect causal relationships. Finally, the sample was predominantly composed of White (94.8%), and the high prevalence of alcohol consumption (over 90%) may have influenced the neurological outcomes. Therefore, caution should be exercised when generalizing these findings to other racial or cultural groups.

Nonetheless, the findings clearly indicate that individuals who do not engage in regular physical activity are at a higher risk of developing neurological diseases. These findings are especially relevant for populations with low PAV levels, including old individuals, those with high body mass index (BMI), and sedentary individuals. Future research should prioritize large-scale prospective cohort studies to validate these results. In summary, physical activity is significantly associated with a reduced risk of neurological diseases, with the risk exhibiting a non-linear relationship with exercise intensity and volume. Excessive exercise may paradoxically increase disease risk. Physical activity demonstrates protective effects against various specific neurological conditions. The study also highlights the influence of gender, age, and activity levels on disease susceptibility, providing a scientific basis for the development of targeted prevention strategies.

## 5 Conclusion

Increased levels of physical activity are associated with a reduced risk of numerous nervous system diseases, with optimal protection observed in this study. It is advocated that moderate-to-vigorous physical activity need to be considered when referring to daily activity as it is the protective factor for neurological disorders, particularly among high-risk populations such as older adults and those leading sedentary lifestyles. Further mechanistic studies and randomized controlled trials are necessary to confirm the causal relationships suggested by this research. And disease-specific risks and non-linear patterns should be taken into account when formulating future public health recommendations.

## Data Availability

The datasets presented in this study can be found in online repositories. The names of the repository/repositories and accession number(s) can be found at: http://www.ukbiobank.ac.uk/.
